# Determinants of health-related quality of life in Iranian adults: evidence from a cross-sectional study

**DOI:** 10.4178/epih.e2017038

**Published:** 2017-08-15

**Authors:** Satar Rezaei, Mohammad Hajizadeh, Ali Kazemi, Masoud Khosravipour, Farid Khosravi, Shahab Rezaeian

**Affiliations:** 1 Research Center for Environmental Determinants of Health, Kermanshah University of Medical Sciences, Kermanshah, Iran; 2 School of Health Administration, Faculty of Health, Dalhousie University, Halifax, Canada; 3 Student Research Committee, Kermanshah University of Medical Sciences, Kermanshah, Iran

**Keywords:** Quality of life, Adult, Visual analog scale, Iran

## Abstract

**OBJECTIVES:**

This study aimed to measure the level and determinants of health-related quality of life (HRQoL) in adults in Kermanshah, a city in the western region of Iran.

**METHODS:**

Convenience sampling was employed to obtain a sample of 998 adults aged 18 years and older (646 males and 352 females) in the city of Kermanshah. A 2-part self-administered questionnaire was used to collect data over the period between March 1 and May 30, 2017. The first part was designed to collect information on socio-demographic characteristics, socioeconomic status, and lifestyle factors (10 items). The second part consisted of the EuroQoL 5-dimensions (EQ-5D) EuroQoL-3-level and the EuroQoL visual analog scale (EQ-VAS) questions. A multiple linear regression model was used to determine the factors associated with the EQ-5D index and EQ-VAS score among study participants.

**RESULTS:**

The mean values for the EQ-5D index and the EQ-VAS score were 0.74 (standard deviation [SD], 0.19) and 80.9 (SD, 16.5), respectively. The highest percentage of self-reported problems (‘some’ and ‘severe’ problems) across the 5 dimensions of the EQ-5D index were associated with the dimensions of anxiety/depression (35.3%) and pain/discomfort (32.9%). The percentage of self-reported problems for the dimensions of usual activities, mobility, and self-care were 19.0, 12.8, and 8.9%, respectively. Our regression analyses indicated that there were statistically significant positive associations between being physically active, monthly household income per capita, and post-secondary education and the EQ-5D index and EQ-VAS score. In contrast, negative associations were found between older age, being married, having a chronic disease, and smoking and the EQ-5D index and EQ-VAS score. A negative association was also found between being uninsured and the EQ-5D index.

**CONCLUSIONS:**

Our findings suggest that interventions aiming to improve physical activity, to prevent chronic diseases, and to reduce the smoking rate among adults living in the city of Kermanshah may improve their HRQoL.

## INTRODUCTION

Ensuring an equal distribution of health-related quality of life (HRQoL) across different social groups and regions is important for policymakers. Hence, measuring and monitoring HRQoL in the general population is critical for developing appropriate health policy interventions to reduce social and geographical inequality in HRQoL throughout society [1,2]. Population-level studies that aim to measure the level and determinants of quality of life (QoL) can potentially provide valuable information about HRQoL across different socio-demographic and socioeconomic groups [2].

The level and determinants of HRQoL in the general population have been well documented in developed countries such as Sweden [1], Netherlands [3], Norway [4], the UK [5], the US [6], and Japan [7]. There are, however, few studies on HRQoL in developing countries. To date, some studies in Iran have investigated QoL in specific population groups. For example, Tajvar and colleagues [8] examined HRQoL and its determinants in the elderly population of Tehran, Iran. Other studies have investigated HRQoL in the elderly in different regions of Iran [9,10]. Some studies have also focused on QoL in specific patient groups, such as patients with multiple sclerosis [11], diabetes [12,13], thalassemia [14], coronary artery disease [15], and tuberculosis [16]. Another study by Karyani et al. [17] examined HRQoL and it determinants among individuals with health insurance coverage in Tehran, Iran. That study showed that factors such as sex, age, educational attainment, income, having a chronic disease, and body mass index (BMI) had significant impacts on the QoL of insured people. Furthermore, a study by Ghafari et al. [18] likewise suggested that sex, place of residence, and educational attainment had a significant influence on the QoL of individuals living in the city of Qom, Iran.

Thus, to date, only a limited number of studies have investigated HRQoL in Iran. There is even less evidence on the level and determinants of HRQoL in the general population of Iran [18,19]. In this study, we aimed at filling this gap by investigating HRQoL in the city of Kermanshah, located in the western part of Iran. We used the EuroQoL 5-dimensions scale (EQ-5D; one of the popular general instruments for measuring HRQoL) and the EuroQoL visual analog scale (EQ-VAS) to examine the level and determinants of HRQoL in adults in Kermanshah. Understanding the determinants of HRQoL in the general population is important for designing and implementing policies to promote and improve the QoL of Iranian adults.

## MATERIALS AND METHODS

### Study setting

This study was conducted in the city of Kermanshah, the capital of Kermanshah Province. This province consists of 14 counties and is in the western region of Iran. The province is bordered by Kurdistan Province to the north, Hamadan and Lorestan Provinces to the east and Ilam Province to the south. It is also bordered by Iraq to the west. The total population of the province was estimated to be approximately 2 million in 2015.

### Study population and sampling method

This was a cross-sectional study, carried out from March to May 2017 that measured HRQoL and its main determinants in adults aged 18 years and older in Kermanshah, Iran. We calculated the sample size using the following sample size calculation formula:

$$n=\frac{Z^{2}\delta^{2}}{d^{2}}$$

Where *n* indicates the required sample size at a 95% level of significance (*z*= 1.96), *δ* denotes the standard deviation (SD) of the HRQoL scores, and *d* is the degree of precision of the mean of HRQoL scores, which was set at 0.015. The mean and SD values were obtained from a previous study conducted in Iran among the general population [18]. To increase the generalizability of the findings of this study, we added 25% more adults to the required sample size of 825, and sampled 1,035 adults in the study. A total of 998 adults (646 males and 352 females) participated in the study, with a response rate of 96%. Samples were selected in 2 stages. First, the city of Kermanshah was divided into northern, southern, western, eastern, and central areas. Then, equal samples from each area were selected by convenience sampling.

### Instrument and measurement

A self-administered questionnaire with 2 parts was used for data collection. The first part included questions related to the sociodemographic characteristics (age, sex, marital status), socioeconomic status (household monthly income, educational attainment, having health insurance), and lifestyle factors (smoking behavior, physical activity, BMI, and having a chronic disease) of the study participants. The second part included a validated Iranian version of the EQ-5D–3level (EQ-5D–3L) questionnaire [17]. The EQ-5D–3L was introduced by the EuroQoL Group. As a standard questionnaire for measuring HRQoL, the EQ-5D–3L consists of 5 dimensions (mobility, self-care, usual activity, pain/discomfort, and anxiety/depression), with 3 response levels for each dimension (no problem, some problem, or extreme problem). The EQ-5D–3L also includes the EQ-VAS, which allows participants to rate their health status on a scale from 1 to 100 [20]. The Iranian value set for EQ-5D–3L health states, calculated by the VAS method in a recent study conducted by Goudarzi et al. [21], was used to compute the HRQoL of participants.

### Statistical analysis

We used the Kolmogorov-Smirnov test to examine the normality of the data distribution. The t-test and analysis of variance were used to identify significant differences in the mean EQ-5D index and EQ-VAS score according to explanatory variables. The EQ-5D index and EQ-VAS score were used as outcome variables. We used age, sex, marital status, monthly household income per capita, educational attainment, having health insurance, physical activity, smoking behavior, BMI, and having a chronic disease as explanatory variables. The BMI was computed as weight in kilograms divided by height in meters squared (kg/m^2^ ). Following World Health Organization recommendations, participants were classified into 3 groups based on their BMIs: normal weight (< 25 kg/m^2^), overweight (25-30 kg/m^2^), and obese (≥ 30 kg/m^2^). Respondents were classified into 3 groups based on their smoking behavior: current smokers (if they smoked at least one cigarette per day), former smokers (if they smoked regularly or occasionally in the past), and never smokers (if they never smoked or had smoked fewer than 100 cigarettes in their lifetime). Multiple linear regression was employed to determine the main determinants of the EQ-5D index and EQ-VAS score. Variance inflation factors and the tolerance index were used to check for collinearity among the explanatory variables. The p-values< 0.05 were considered to indicate statistical significance. All analyses were performed using the statistical package Stata version 14.2 (StataCorp, College Station, TX, USA).

### Ethical issues

This study was approved by the Ethics Committee of the Deputy of Research of the Kermanshah University of Medical Sciences (KUMS.REC.1396.240). Participants were assured that their personal information and answers would remain confidential.

## RESULTS

The average age of the participants was 32.2 years, with a SD of 12.4 years. Most of the participants (53.2%) fell into the low-income category (monthly household income per capita < 10 million Iranian rials), 28.1% were current smokers, and 61.2% were physically active. Of the participants, 56.5% had a normal weight, while the remaining 43.5% were either overweight or obese. The mean values of the EQ-5D index and EQ-VAS score were 0.74 (SD, 0.19) and 80.9 (SD, 16.5), respectively. We observed a positive significant association between the EQ-5D index and EQ-VAS score (r= 0.63; p< 0.001) in the sample. Table 1 presents the average EQ-5D index and EQ-VAS score in the study population according to socio-demographic characteristics, socioeconomic status, and lifestyle factors.

**Figure 1.**
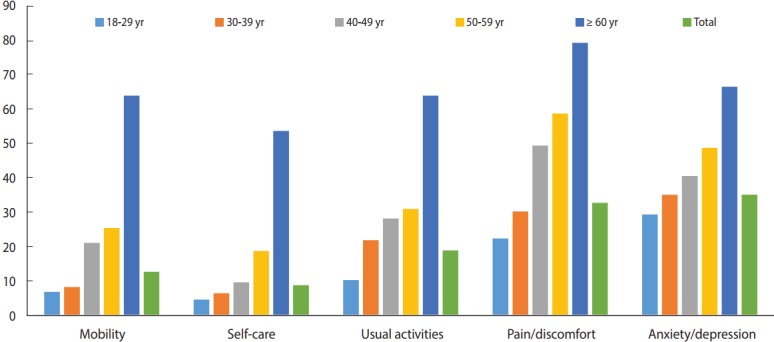
Percentage of respondents who reported problems (‘some’ or ‘severe’) among the adult population by age group in Kermanshah, Iran, 2017.

As shown in Table 1, statistically significant differences were found in the EQ-5D index and EQ-VAS score by age group, marital status, BMI, smoking behavior, physical activity, household monthly income per capita, educational attainment, having health insurance, and having a chronic disease (p< 0.05). While we did not observe a significant difference in the EQ-5D index according to sex (p= 0.83), the association between sex and the EQ-VAS score was statistically significant (p= 0.03). Figure 1 and Table 2 present descriptive statistics for participants’ detailed responses to the 5 EQ-5D dimensions by age groups. The highest percentage of reported problems (‘some’ or ‘severe’) across the 5 dimensions was associated with anxiety/depression (35.3%), followed by pain/discomfort (32.9%), usual activities (19.0%), mobility (12.8%), and self-care (8.9%). None of the participants reported having ‘severe’ problems with mobility, self-care, or usual activities (Figure 1 and Table 2).

The results of multiple linear regression were presented in Table 3. The value of the variance inflation factors was 2.54, indicating no multicollinearity among the independent variables. The results suggested that the independent variables explained approximately 31.00 and 41.50% of the variance in the EQ-5D index and EQ-VAS score, respectively. Statistically significant positive associations were found between being physically active, household monthly income per capita, and post-secondary educational attainment and the EQ-5D index and EQ-VAS score. In contrast, significant negative associations were observed between older age, being married, having a chronic disease, and being a current or former smoker and the EQ-5D index and EQ-VAS score. No significant association was found between sex and the EQ-5D index. We only observed a negative association between being overweight and the EQ-VAS score. In addition, a negative association was found between being uninsured and the EQ-5D index.

## DISCUSSION

Measuring HRQoL among the general population is an important issue for health policymakers and is critical for the development of appropriate interventions to improve the QoL of individuals within society. This study used the EQ-5D–3L questionnaire to measure HRQoL and its main determinants among the general population of the city of Kermanshah, Iran, in 2017. Pain/discomfort and anxiety/depression were the most common health problems among the general population in Kermanshah. These findings are similar to the findings of previous studies in Iran [17] and other countries [22,23]. One of the potential factors contributing to the higher proportion of these 2 health problems in Kermanshah is the high unemployment rate of 23.2% in this province [24], which leads to a lower QoL among adults living in this city.

We found that having a chronic disease was associated with poor HRQoL, based on both the EQ-5D index and the EQ-VAS score. Several studies have indicated that chronic diseases such as hypertension [25], diabetes [26], coronary heart disease [15], tuberculosis [16], multiple sclerosis [27], cancers [28], and allergic diseases [29] reduce HRQoL among adults. A study by Karyani et al. [17] showed that chronic disease was an important determinant of HRQoL in Iran. That study suggested that the prevention and management of chronic diseases should be a priority for improving HRQoL in Iran. Similar to the study by Karyani et al. [17], our study indicated that having health insurance was significantly positively related with the EQ-5D index. The positive impact of having health insurance coverage on HRQoL can be explained by improved access to appropriate and timely healthcare services.

We also found that risky behaviors such as smoking and being physically inactive were significantly associated with a poor EQ-5D index and EQ-VAS score. The current literature [30] suggests that individuals who engage in risky behaviors pay less attention to their health, contributing to the onset of chronic diseases that, in turn, reduce HRQoL among this population group. Studies from other countries have demonstrated that ceasing risky behaviors leads to a higher HRQoL among the adult population. For example, a study in Spain [31] reported that former smokers had better health scores than their smoking counterparts. As a result, educational and preventive programs are required to reduce risky behaviors among these groups within society. Although being obese has been shown to have a negative influence on HRQoL [26], we did not observe a significant relationship between obesity and HRQoL in our study.

Moreover, our findings showed that aging had a negative effect on HRQoL. Iran’s population is aging [32], which presents challenges for policymakers to improve the QoL of older adults in Iran. Aghamolaei et al. [10] examined the HRQoL of older adults in Bandar Abas (the southern region of Iran) and reported that it was lower in older adults. Similarly to that study [10], we found that higher levels of educational attainment were associated positively with HRQoL among adults. In addition, we found a significant positive relationship between monthly household income per capita and HRQoL in the adult population. Income, as an indicator of socioeconomic status, is associated with healthcare-seeking behaviors, which can affect the QoL of adults. In fact, previous studies [33,34] have already demonstrated higher utilization of healthcare services among higher socioeconomic status groups in Iran.

Our study is subject to some limitations. First, we were unable to establish causal relationships between the explanatory and dependent variables due to the cross-sectional design of this study. Second, the results related to some of the self-reported variables, such as monthly household income per capita, should be interpreted with caution. Lastly, the use of a non-random sampling method in the study may limit the generalizability of the findings. Even considering these caveats, the results of our study suggest that health policymakers should focus on the effects of the major lifestyle and socioeconomic factors affecting HRQoL to improve the QoL among the general population in Iran. Nonetheless, further studies are required to determine the most effective interventions to improve the HRQoL of Iranian adults.

In summary, our study demonstrated the effects of socio-demographic characteristics, socioeconomic status, and lifestyle factors on HRQoL among the general population of adults living in Kermanshah, western Iran. The results suggested that interventions aiming to increase physical activity, to prevent and manage chronic diseases, and to reduce the smoking rate among Iranian adults may improve their HRQoL.

## Figures and Tables

**Table 1.** The average EQ-5D index and EQ-VAS scores by socio-demographic characteristics, socioeconomic status, and lifestyle factors in the study population

Variables	Frequency (%)	EQ-5D	p-value	EQ-VAS	p-value
Age (yr)					
18-44	771 (77.2)	0.78 ± 0.16	<0.001^1^	83.4 ± 15.1	<0.001^1^
≥ 45	227 (22.8)	0.63 ± 0.23		72.4 ± 18.4	
Sex					
Male	646 (64.7)	0.74 ± 0.19	0.83^1^	80.1 ± 16.1	0.03^1^
Female	352 (35.3)	0.75 ± 0.19		82.4 ± 17.2	
Marital status					
Never married	471 (47.2)	0.79 ±0.15	<0.001^2^	84.7 ± 14.6	<0.001^2^
Married	480 (48.1)	0.71 ±0.21		78.0 ± 16.9	
Divorced and widowed	47 (4.7)	0.61 ± 0.24		72.3 ± 21.5	
Monthly household income per capita (IRR)^3^					
Low (<10 million)	531 (53.2)	0.74 ± 0.19	<0.001^2^	80.6 ± 18.3	<0.001^2^
Middle (10-20 million)	344 (34.5)	0.73 ± 0.20		79.0 ± 14.1	
High (>2 million)	123 (12.3)	0.79 ± 0.15		87.6 ± 13.2	
Educational attainment					
Illiterate	33 (3.3)	0.51 ±0.26	<0.001^2^	60.0 ± 19.7	<0.001^2^
Primary and secondary	459 (46.0)	0.72 ± 0.20		79.1 ± 16.3	
Post-secondary	506 (50.7)	0.78 ± 0.15		83.9 ± 15.2	
Health insurance					
Yes	828 (83.0)	0.75 ± 0.19	0.001^1^	81.3 ± 15.5	0.001^1^
No	170 (17.0)	0.70 ± 0.20		79.2 ± 20.8	
Smoking behavior					
Current	280 (28.1)	0.70 ± 0.22	<0.001^2^	73.2 ± 15.5	<0.001^2^
Former	56 (5.6)	0.67 ± 0.21		72.7 ± 17.5	
Never	662 (66.3)	0.77 ± 0.17		84.7 ± 15.5	
Physical activity					
Active	610 (61.2)	0.80 ± 0.14	<0.001^2^	86.4 ± 12.2	<0.001^2^
Moderately active	283 (28.3)	0.69 ± 0.20		75.7 ± 15.8	
Inactive	105 (10.5)	0.56 ± 0.25		63.1 ± 22.3	
Body mass index					
Normal	564 (56.5)	0.76 ± 0.18	<0.001^2^	83.3 ± 15.7	<0.001^2^
Overweight	367 (36.8)	0.73 ± 0.20		78.1 ± 16.7	
Obese	67 (6.7)	0.67 ± 0.24		76.7 ± 19.1	
Chronic diseases					
Yes	131 (13.1)	0.56 ± 0.22	0.001^1^	63.5 ± 18.9	0.001^1^
No	867 (86.9)	0.77 ± 0.17		83.5 ± 14.4	

Values are presented as mean±standard deviation.

EQ-5D, EuroQoL 5-dimensions; EQ-VAS, EuroQoL visual analog scale; QoL, quality of life; IRR, Iranian rial.

1 Statistical significance of differences calculated using the t-test.

2 Statistical significance of differences calculated using analysis of variance.

3 One US dollar = 35,000 IRR in 2017.

**Table 2.** Detailed responses of participants to the 5 dimensions of the EQ-5D according to ahe troup in Kermanshah, western Iran, 2017

EQ-5D	Level (problem)	Age (yr)					Total
		18-29	30-39	40-49	50-59	≥60		
Mobility	No	93.0	91.6	78.8	74.4	35.9	87.2
	Some	7.0	8.4	21.2	25.6	64.1	12.8
	Severe	0.0	0.0	0.0	0.0	0.0	0.0
Self-care	No	95.3	93.4	90.3	81.1	46.2	91.1
	Some	4.7	6.6	9.7	18.9	53.8	8.9
	Severe	0.0	0.0	0.0	0.0	0.0	0.0
Usual activities	No	89.6	78.0	71.7	68.9	35.9	81.0
	Some	10.4	22.0	28.3	31.1	64.1	19.0
	Severe	0.0	0.0	0.0	0.0	0.0	0.0
Pain/discomfort	No	77.5	69.6	50.4	41.1	20.5	67.1
	Some	21.6	30.4	48.7	57.8	71.8	31.9
	Severe	0.9	0.0	0.9	1.1	7.7	1.0
Anxiety/depression	No	70.5	64.8	59.3	51.1	33.3	64.7
	Some	28.2	33.0	37.2	45.6	56.4	33.0
	Severe	1.3	2.2	3.5	3.3	10.3	2.3

Values are presented as %.

EQ-5D, EuroQoL 5-dimensions; QoL, quality of life.

**Table 3.** Multiple linear regression results of the association between explanatory variables and the EQ-5D index and EQ-VAS score

Explanatory variables	Dependent variables					
	EQ-5D			EQ-VAS						
	Coefficient	p-value	95% CI	Coefficient	p-value	95% CI
Age (yr)						
18-44	Reference			Reference		
≥ 45	-0.07	0.00	-0.09, -0.04	-3.40	0.005	-5.70, -1.02
Sex						
Male	Reference			Reference		
Female	0.01	0.28	-0.01,0.04	1.55	0.12	-0.41, 3.53
Marital status						
Never married	Reference			Reference		
Married	-0.02	0.07	-0.04,0.00	-2.20	0.02	-4.20, -0.29
Divorced and widowed	-0.07	0.006	-0.12, -0.02	-2.70	0.20	-6.80, 1.40
Monthly household income per capita (IRR)^1^						
Low (<10 million)						
Middle (10-20 million)	0.012	0.33	-0.01, 0.04	1.40	0.15	-0.53, 3.30
High (>2 million)	0.06	0.00	0.03, 0.01	8.54	0.00	5.80, 11.30
Educational level						
Illiterate	Reference			Reference		
Primary and secondary	0.04	0.15	-0.02, 0.10	5.50	0.02	0.66, 10.40
Post-secondary	0.07	0.04	0.00, 0.13	5.40	0.03	0.41, 10.40
Health insurance						
Yes	Reference			Reference		
No	-0.04	0.01	-0.06, -0.01	-1.33	0.24	-3.60, 0.90
Smoking behavior						
Never	Reference			Reference		
Former	-0.06	0.00	-0.10, -0.02	-8.80	0.00	-12.40, -5.20
Current	-0.03	0.00	-0.06, -0.01	-8.80	0.00	-10.90, -6.70
Physical activity						
Inactive	Reference			Reference		
Moderately active	0.09	0.00	0.05, 0.13	15.9	0.00	13.00, 18.70
Active	0.15	0.00	0.12, 0.19	8.90	0.00	5.90, 11.90
Body mass index						
Normal	Reference			Reference		
Overweight	-0.002	0.87	-0.02, 0.02	-1.90	0.03	-3.70, -0.19
Obese	-0.02	0.31	-0.06, 0.02	-2.10	0.21	-5.50, 1.20
Chronic diseases						
Yes	Reference			Reference		
No	0.11	0.00	0.08, 0.15	11.3	0.00	8.70, 13.90
R-squared (%)		31.00			41.50	
Adjusted R-squared (%)		29.90			40.00	
Prob > F		0.00			0.00	

EQ-5D, EuroQoL 5-dimensions; EQ-VAS, EuroQoL visual analog scale; QoL, quality of life; IRR, Iranian rial; CI, confidence interval.

1 One US dollar = 35,000 IRR in 2017.
